# IgA nephropathy-associated breast milk B cell alterations

**DOI:** 10.3389/fimmu.2026.1784598

**Published:** 2026-05-08

**Authors:** Katerina Zachova, Alica Cutkova, Petr Kosztyu, Yukako Ohyama, Kazuo Takahashi, Josef Zadrazil, Jiri Orsag, Nadezda Petejova, Jiri Mestecky, Milan Raska

**Affiliations:** 1 Department of Immunology, Faculty of Medicine and Dentistry, Palacky University Olomouc, Olomouc, Czechia; 2 Department of Immunology, University Hospital Olomouc, Olomouc, Czechia; 3 Department of Biomedical Molecular Sciences, School of Medicine, Fujita Health University, Nagoya, Japan; 4 Department of Internal Medicine III Nephrology, Rheumatology and Endocrinology, University Hospital Olomouc, Olomouc, Czechia; 5 Department of Internal Medicine III Nephrology, Rheumatology and Endocrinology, Faculty of Medicine and Dentistry, Palacky University Olomouc, Olomouc, Czechia; 6 Department of Internal Medicine and Cardiology University Hospital Ostrava and Faculty of Medicine University of Ostrava, Ostrava, Czechia; 7 Department of Microbiology, University of Alabama at Birmingham, Birmingham, AL, United States; 8 Department of Medicine, University of Alabama at Birmingham, Birmingham, AL, United States

**Keywords:** B cells, breast milk, Epstein–Barr virus, galactose deficient IgA1, IgA nephropathy, migrating pre-plasma cells, mucosal immunity

## Abstract

**Background:**

IgA nephropathy (IgAN) is a mucosal immune-associated disease characterized by the overproduction of galactose-deficient IgA1 (Gd-IgA1) and formation of nephritogenic immune complexes. Epstein–Barr virus (EBV), which preferentially infects IgA^+^ B cells, has been implicated in IgAN pathogenesis, although its role remains unclear. Given mucosa involvement, we characterized breast milk B cells as available representatives of mucosal tissue to better understand the pathogenesis of IgAN.

**Methods:**

We performed a phenotypic analysis of B cells (CD19^+^), plasmablasts (CD38^+^), and CD138^+^ cells (representing migrating pre-plasma cells, CPC) in the breast milk of IgAN and healthy mothers (HC). EBV infection of cells was detected by using EBV-encoded RNA (EBER). B cell differentiation, IgA/Gd-IgA1 expression, and homing-related markers were characterized using complementary cytometric and microscopic approaches.

**Results:**

Breast milk from mothers with IgAN contains a significantly higher proportion of EBER^+^ B cells compared with HC. Moreover, the proportion of EBER^+^ CPCs in the breast milk of IgAN mothers is significantly higher than in HC. B cells present in the breast milk of mothers with IgAN exhibit a higher expression of the mucosal homing receptor CCR9 compared to B cells from HC. IgA^+^ B cells from healthy mothers exhibit a higher overall frequency of surface Gd-IgA1 expression and lack CD138 marker and thus could be classified as memory B cells or plasmablasts considering their class-switched phenotype. In contrast, breast milk from IgAN mothers was enriched for Gd-IgA1^+^ CD138^+^ CPC cells, indicating a shift toward terminally differentiated antibody-producing cells. These findings suggest disease-associated alterations in B cell differentiation and compartmentalization rather than increased mucosal Gd-IgA1 production *per se*.

**Conclusion:**

Despite the limited number of analyzed samples, we detected interesting differences in B cells in the breast milk of IgAN mothers and HC. The analysis of B cell populations in the breast milk of IgAN mothers indicates EBV-associated B cell dysregulation. The enrichment of EBV^+^ CPC and Gd-IgA1^+^ CPC in the breast milk of IgAN mothers agrees with a former model proposing aberrant mucosal B cell differentiation and trafficking involvement in IgAN pathogenesis.

## Introduction

Epstein–Barr virus (EBV) has been associated with a wide range of human diseases, including lymphoproliferative disorders, epithelial malignancies, and several autoimmune conditions (1–5). Immunoglobulin A nephropathy (IgAN), one of the most common primary glomerulonephritis, is an autoimmune disease characterized by mesangial deposition of immune complexes containing polymeric galactose-deficient IgA1 (Gd-IgA1). This leads to renal damage which progresses in 40% of patients to end-stage renal disease. The hinge region of IgA1, enriched in *O*-glycans, renders Gd-IgA1 an autoantigen for IgG and IgA autoantibodies, implicating dysregulation of the IgA system in disease pathogenesis (6, 7).

The marked geographic variation in IgAN prevalence—high in East Asia, Europe, Australia, and North America, but low in central Africa and among African Americans—suggests that environmental factors interact with host genetics to influence disease susceptibility (7). EBV is a strong candidate environmental trigger, as it preferentially infects IgA^+^ B cells and promotes their differentiation into IgA1-secreting plasma cells, including cells producing Gd-IgA1 in patients with IgAN. Epidemiological observations further support this association: primary EBV infection typically occurring in early childhood in African populations coincides with the immaturity of the IgA system, potentially limiting viral access to IgA^+^ B cells, which is later limited by EBV-elicited specific immune responses. On the other hand, primary EBV infection during adolescence, typical for White populations, preferentially targets surface IgA^+^ B cells (7, 8).

Despite this biological plausibility, *in vivo* characterization of EBV-infected cells has been limited by the absence of distinguishing surface markers. Detection of EBV-positive CD19^+^ B cells in the blood and their phenotypic characterization could be based on the combination of *in situ* hybridization (ISH) for EBV-encoded RNA with flow cytometry (8, 9). Because the stringent conditions required for ISH preclude simultaneous staining for Gd-IgA1, parallel analyses were performed to characterize EBV infection, migration profile of CD19^+^ cells, and IgA-related phenotypes in breast-milk-derived cells from female patients with IgAN and healthy controls (8–10).

The terminology describing antibody-producing cells developed across literature through time. Earlier work distinguished preferentially discrete stages such as plasmablasts and plasma cells, whereas recent literature suggests that B-cell maturation is a continuous process progressing from B cells through activated cells and plasmablasts to circulatory precursors of plasma cells and, ultimately, plasma cells (11, 12). Plasmablasts are generally CD38^+^ and CD138^-^, while plasma cells are CD138^+^. However, analyses of migrating plasma cell phenotype, such as those identified in the peripheral circulation of healthy subjects, have identified a CD19^+^CD38^+^CD138^+^ subpopulation that morphologically does not resemble the fully mature classical antibody-secreting cells (plasma cells). Circulatory plasma cells are thought to represent an intermediate stage termed circulating or migrating pre-plasma cells (CPCs), transiently traveling to survival niches for final maturation into plasma cells. Thus, we decided to use the term “circulatory or migrating pre-plasma cells” abbreviated as “CPCs” throughout the entire manuscript.

Here we tested the hypothesis that EBV-driven alterations in mucosal CD19^+^ B cell populations contribute to IgAN pathogenesis. Building on our previous findings in peripheral blood mononuclear cells (9), we conducted an *in vivo* phenotypic analysis of EBV-infected B cells in breast milk from mothers with IgAN and healthy controls. Because immune cells in breast milk closely reflect mucosal immune responses, this approach allows a direct assessment of EBV-associated perturbations in the mucosal B cell compartment. In parallel, we identify disease-specific differences in IgA^+^, Gd-IgA1^+^, and CD138^+^ cell populations in the breast milk of mothers with IgAN and healthy controls, indicating alterations in mucosal IgA-associated immune responses in IgA nephropathy.

## Materials and methods

### Reagents

All chemicals, unless otherwise specified, were from Thermo Fisher Scientific (MA, USA).

### Study subjects

This study was conducted in accordance with the principles of the Declaration of Helsinki. The study subjects signed an informed consent approved by the Ethical Committees of University Hospital Olomouc, Olomouc, Czech Republic (no.: 121/14) in August 21, 2014.

The IgAN mothers were recruited at the Nephrology, Rheumatology, and Endocrinology Department, University Hospital Olomouc. The diagnosis of IgAN had been based on staining for IgA as the dominant or codominant Ig in the mesangial immune deposits by routine immunofluorescence microscopy of clinically indicated kidney biopsies, in the absence of clinical or laboratory features of SLE nephritis, IgA vasculitis, or liver disease. Baseline clinical data, including age, blood pressure, serum creatinine level, eGFR, urinary albumin/creatinine ratio (ACR), 24-h proteinuria, hematuria, and treatment with angiotensin-converting enzyme (ACE) inhibitor or angiotensin receptor blocker (ARB), were obtained from the review of medical records. The health-control group consisted of breast-feeding mothers recruited from Department of Immunology, University Hospital Olomouc. The clinical and biochemical characteristics of the study participants are presented in **Table 1**. During pregnancy and breastfeeding, the IgAN patients were not on corticosteroid therapy; the disease state was assessed as non-progressive (stable). ACE inhibitors are contraindicated during pregnancy and breastfeeding. The IgAN mothers were sampled twice—first for EBV analyses and then for Gd-IgA and fluorescent microscopy studies. The healthy controls were sampled three times—first for optimization experiments, then for EBV determination, and lastly for Gd-IgA studies and fluorescent microscopy.

**Table 1:** Clinical and biochemical characteristics of the study subjects.

Characteristics	IgAN	Healthy
Number of subjects	3	3
Age (years)	34.67 ± 14.57	30.33 ± 2.517
Time since diagnosis (years)	5.00 ± 5.29	ND
Blood pressure (mean values)	123.3/73.33	120/80
Serum IgA concentration (mg/mL)	2.29 ± 1.32	1.25 ± 0.51
Gd-IgA1 (µg/mL)	4.93 ± 1.6	4.31 ± 2.25
eGFR (mL/min per 1.73 m^2^)	1.4 ± 0.17	ND
Serum creatinine (μmol/L)	77.33 ± 12.74	ND
Serum urea (mmol/L)	3.9 ± 0.6	ND
Proteinuria (g/day)	0.35 ± 0.25	ND
Urinary albumin/creatinine (ACR; mg/mmol)	14.17 ± 19.89	ND
Hematuria (Ery/μL)	137.7 ± 226.4	ND
Disease activity^a^	non-progressive	
Stabilized/slow progression/progression	3/0/0	

a Disease progression was based on a decrease in eGFR of more than 10% per year (10).

### Baseline characteristics of the study population

### Isolation of PBMCs from breast milk

The entire isolation was performed at 4 °C. Breast milk of 50 mL was centrifuged at 600 × *g* for 15 min at 4 °C (13). Milk fat was removed by using a Pasteur pipette, and when it was extremely thick, it was removed by using a spoon. The skim milk fraction was pipetted into a new tube for further ELISA analyses. The cell pellet was washed twice with phosphate-buffered saline (PBS) and separated by centrifugation at 600 × *g* 15 min at 4 °C.

### Determination of IgA and Gd-IgA1 levels in serum and breast milk

The total level of IgA in serum and breast milk was quantified using ELISA method, following an established protocol (14, 15). The Gd-IgA1 levels were assessed using the 35A12 monoclonal antibody (mAb) (10). ELISA plates (Nunc, Thermo Fisher Scientific) were coated overnight at 4 °C with goat anti-human IgA F(ab’)2 fragments at 1 µg/mL (Jacksons Immunoresearch, PA, USA) and then blocked for 3 h at room temperature using 1% BSA in PBS/Tween20 before adding diluted serum samples or breast milk supernatant (lymphocyte isolation protocol) to the wells. The bound IgA was desialylated by incubation with sialidase A from *Arthrobacter ureafaciens* (ProZyme, Agilent Technologies, CA, USA) at 2 mU/mL in 100 mM PBS (pH 6.0) for 3 h at 37 °C. Gd-IgA1 was then detected using the mouse anti-human Gd-IgA1 IgG3 mAb 35A12 (20 µg/mL in blocking buffer), followed by a rabbit anti-mouse IgG peroxidase-conjugated secondary antibody (1:4,000 dilution in blocking buffer; Merck, Germany). The colorimetric signal was generated with O-phenylenediamine-H_2_O_2_ substrate, stopped, and measured using ELISA reader.

### EBV serology

The serum levels of IgG antibodies specific for EBV antigens VCA and EBNA1 were determined using commercially available ELISA kits (VIDIA, Czech Republic) according to the manufacturer’s instructions. Briefly, serum samples diluted 1:100 in the assay buffer were applied to antigen-coated microtiter plates. Following incubation for 30 min at room temperature, the plates were washed and incubated with horseradish-peroxidase-conjugated secondary monoclonal antibodies. After 1 h, the plates were washed and developed using tetramethylbenzidine (TMB), and optical density was measured at 450 nm.

### Cell staining and *in situ* hybridization

Fc receptors were blocked by using 10% heat-inactivated human serum in PBS at room temperature for 10 min. The cells were washed using PBS and stained with the following surface-marker-specific mAbs conjugated with fluorochromes—anti-CD19-PE-Texas Red, anti-CD19-APC-H7, anti-CD138-PE-cy7, anti-CCR5-PE-cy5, anti-CCR9-PE, anti-CD62L-PE-cy5, anti-CD49d-PE-cy5, anti-β7 integrin-PE-cy7, and anti-CD29-PE (BD Biosciences, NJ, USA) at the manufacturer’s recommended dilution in 5% fetal bovine serum (FBS) in PBS for 30 min at room temperature and in the dark. The cells were washed by using PBS and centrifuged at 600 × *g* for 7 min at room temperature. The cell pellet was fixed by using 4% paraformaldehyde (PFA) for 10 min at room temperature, washed by using room-temperature PBS, and centrifuged at 730 × *g* for 7 min at room temperature. The supernatant was discarded, and the samples were permeabilized by using 50 µL PBS + 0.5% Tween-20 for 10 min at room temperature. Furthermore, 200 µL of adapting buffer contained 31.25 mM NaCl, 6.25 mM Na_2_EDTA, 62.5 mM Tris-HCl, pH 7.5, and 37.5% formamide was added, and the samples were centrifuged at 750 × *g* for 7 min at room temperature. The cell pellet was resuspended in hybridization buffer (10% dextran sulfate, 10 mM NaCl, 30% formamide, 0.1% sodium pyrophosphate, 0.2% polyvinylpyrrolidone, 5 mM Na_2_EDTA, 50 mM TRIS-HCl, pH7.5) containing EBER-1-specific hybridization DNA probes and incubated at 42 °C for 1 h in the dark. The sequences of the three EBER-1 probes used in this protocol are available from (9). All three probes were synthetized and conjugated with Cy3 or Cy5 fluorochromes (Generi Biotech, Czech Republic), depending on the fluorochrome panel used. The cells were washed twice with 0.5% Tween-20 in PBS at 42 °C for 10 and 30 min, respectively, and analyzed by flow cytometry using SP6800 spectral analyzer (SONY Biotechnologies, Japan).

### Cell surface Gd-IgA1 staining

After surface Fc receptor blocking and washing as described above, the cells were incubated with 41 μg/mL of biotin-labeled anti-Gd-IgA1 35A12 mAb in 5% FBS in PBS for 1 h at 37 °C (10, 16). After incubation, the cells were washed, and streptavidin-Pacific Orange, together with mAb (anti-IgA-FITC; Jacksons Immunoresearch), anti-CD19-PE, and anti-CD138-PE-cy7 (BD Biosciences), was added in 5% FBS in PBS for 30 min at room temperature in the dark. After washing, the cells were analyzed by using flow cytometry with the SP6800 spectral analyzer.

### Microscopy

Primary staining was performed in microtubes. After surface Fc receptor blocking and washing as described above, the cells were stained using anti-CD19-PE and anti-IgA-FITC mAb at the manufacturer’s recommended dilution in 5% FBS in PBS for 30 min in the dark. Detection of CPC was performed using anti-CD19-PE and anti-CD138-FITC mAb staining. Detection of Gd-IgA1 was performed after staining using anti-Gd-IgA1-biotin mAb (35A12) for 1 h at 37 °C (17). After intensive washing with PBS, anti-IgA-Pacific Blue (Jacksons Immunoresearch), and streptavidin-FITC, anti-CD19-PE (BD Biosciences) was added to the cells for 30 min at room temperature in the dark. After staining, each cell sample was washed in PBS and cytospinned onto microscope slides at 1,000 × *g* for 5 min. The cells were fixed using 4% PFA for 15 min at room temperature in the dark, and the EBER probe hybridization was performed as described above (18). The cells were analyzed by using the fluorescence microscope DM6 (Leica, Germany).

### Cytometry data processing and statistical analysis

All cytometry data were analyzed using SONY software (SONY Biotechnologies) and FlowJo software (BD Bioscience, CA, USA). Statistical data analysis was performed using GraphPad Prism 8 software (GraphPad Software, CA, USA).

## Results

### Comparison of EBER^+^ cells in breast milk CD19^+^ populations

**Figure 1:**
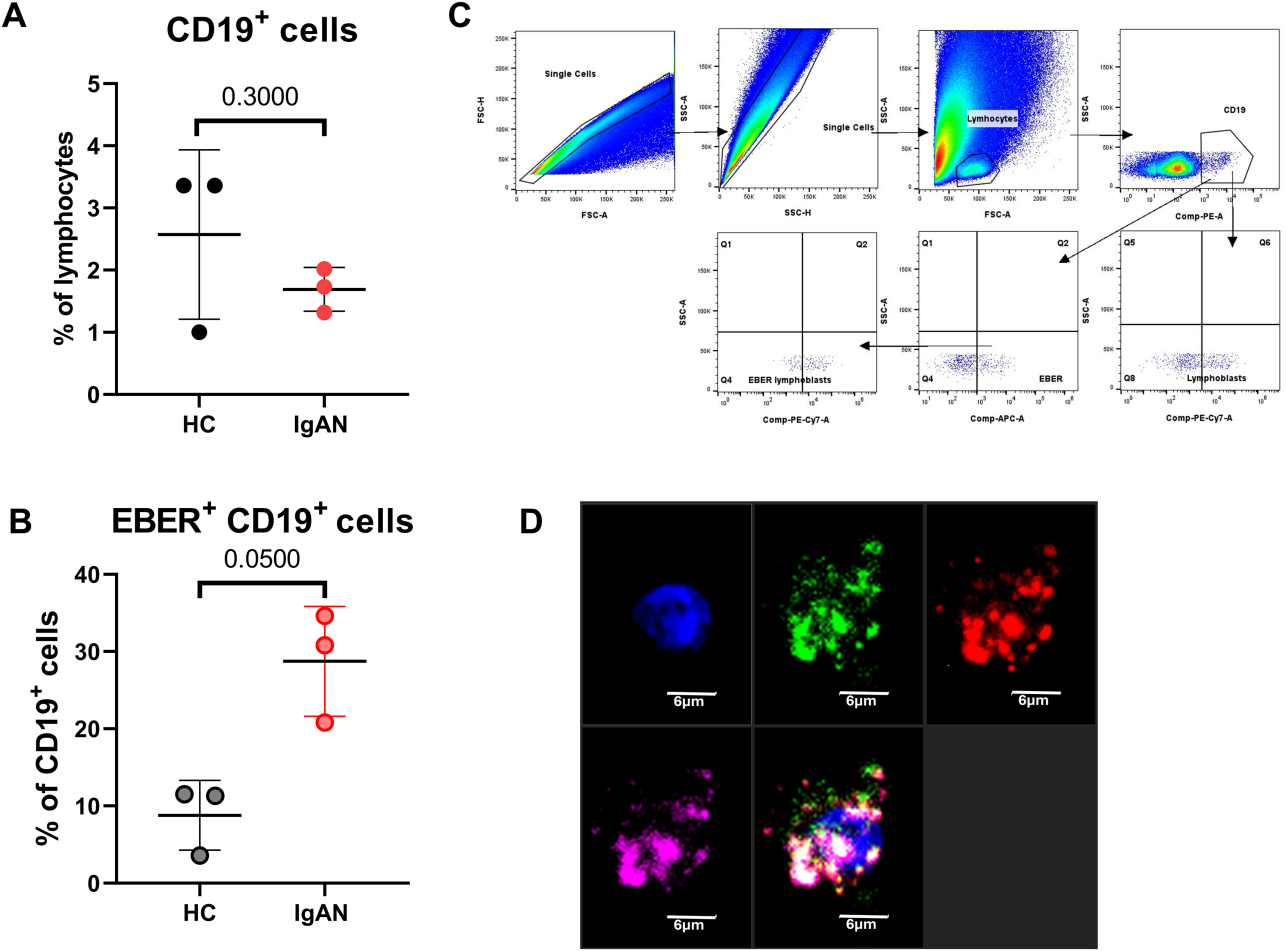
Analysis of CD19^+^ cells in the breast milk of IgAN mothers and healthy controls. **(A)** Differences of CD19^+^ cell populations in the breast milk of IgAN mothers (*n* = 3) and healthy controls (HC) (*n* = 3). **(B)** Differences in EBER^positive^ (EBER^+^) CD19^+^ B cells in the breast milk of healthy mothers and IgAN patients. **(C)** Gating strategy of breast milk cells to detect CD19^+^, EBER^+^, and/or CD138^+^ cells. **(D)** Characterization of IgA^+^ (green), CD19^+^ (red), and EBER^+^ (pink) cells from the breast milk of an IgAN mother. Cell nuclei were stained with DAPI (blue). Significant values were calculated by using non-paired non-parametric Mann–Whitney test.

The levels of CD19^+^ cells in breast milk are lower than in peripheral blood (19). The proportion of milk CD19^+^ cells does not differ between IgAN mothers and healthy mothers, although IgAN mothers exhibit the tendency toward reduced CD19^+^ cells (**Figure 1A**). In contrast, the percentage of EBER^+^ population of all CD19^+^ cells in IgAN mother’s milk is significantly higher (30.80%) than in healthy mothers (11.30%) (**Figure 1B**). The percentage of EBER^+^ population of total CD19^+^ cells in breast milk is higher than in peripheral blood where the values were reported to be 0.4% for IgAN patients and 0.1% for healthy controls (8, 9). Considering that the mothers, both IgAN and healthy controls, were EBV seropositive (**Supplementary Table 1**), these results indicate a distinct migratory potential of EBV-infected cells within an organism in IgAN and healthy controls. The identification of EBER^+^ B cells was based on the elimination of doublets (FSC-A vs. FCS-H, SSC-A vs. SSC-H), size exclusion (FSC vs. SSC), and gating on CD19^+^ population and EBER^+^ population (**Figure 1C**). The percentage of EBER^+^ CD19^+^ cells corresponds linearly with the percentage of CD19^+^ cells irrelevantly to the sampling period during the first 3 months after delivery (**Supplementary Figure 1A**). The results from microscopy show IgA molecules on CD19^+^ EBER^+^ cells (**Figure 1D**).

**Figure 2:**
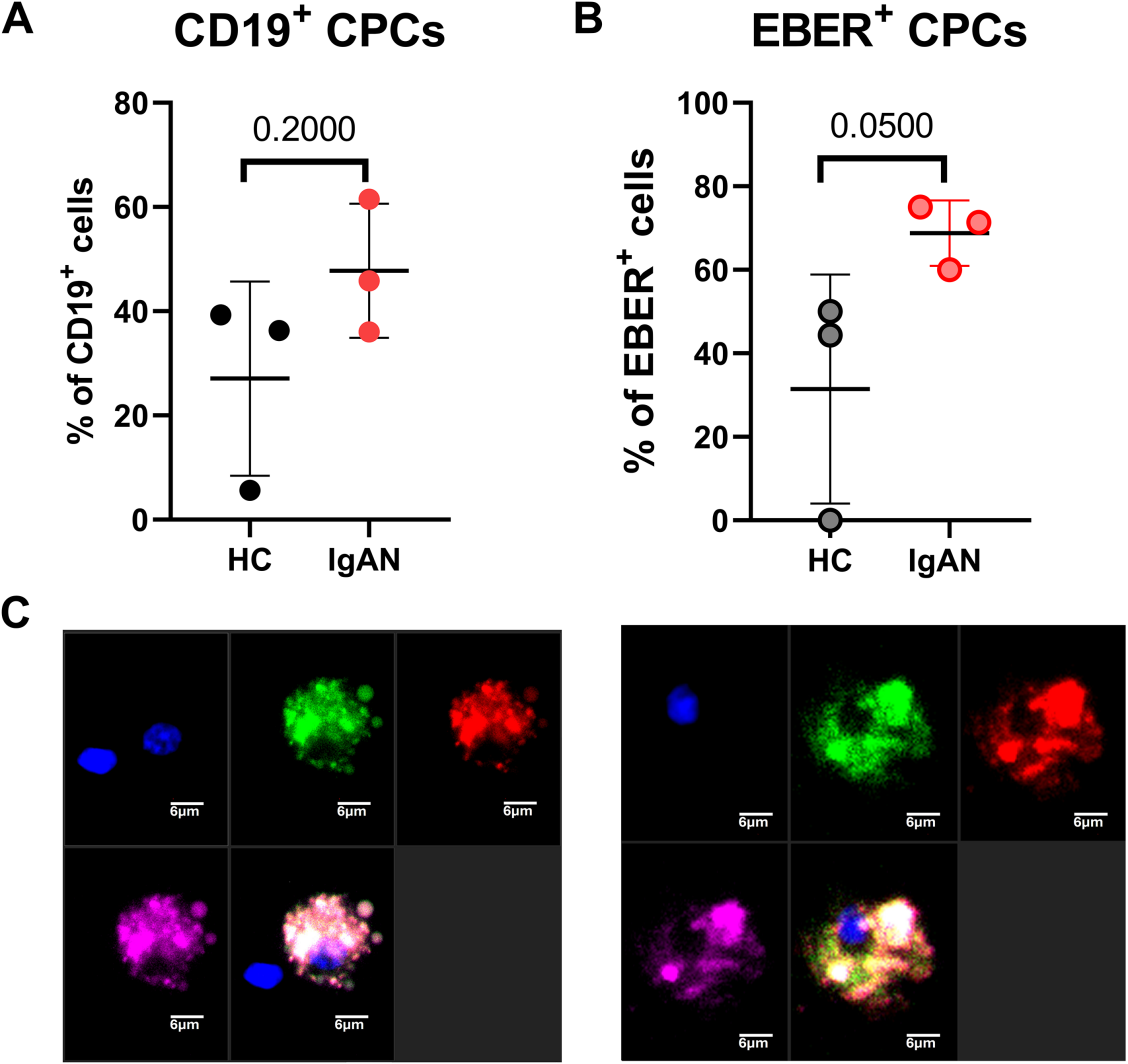
CPC phenotype of CD19^+^ cells in the breast milk of healthy mothers and IgAN patients. **(A)** Population of CD138^+^ cells in CD19^+^ cells. **(B)** EBER^+^ CPC proportions from all EBER^+^ in breast milk. **(C)** Representative microscopic images of two distinct cells positive for CD19 (red), CD138 (green), and EBER (pink) detected in breast milk from an IgAN mother. Cell nuclei were stained with DAPI (blue). Significant values were calculated by using non-paired non-parametric Mann–Whitney test.

Further analysis was focused on migrating pre-plasma cell (CPC) subsets characterized by surface CD19 and CD138 (Syndecan-1) expression. The total percentage of CPC populations in the breast milk of IgAN and healthy mothers does not differ significantly (**Figure 2A**).

The detection of EBER-positive CPC revealed that breast milk from IgAN mothers contains a significantly higher percentage of EBER^+^ CD138^+^ cells (71.4%) than from healthy mothers (44.4%) (**Figure 2B**). The co-expression of surface CD19 and CD138 on these cells was visualized by fluorescent microscopy together with EBER positivity and cell nucleus staining (**Figure 2C**).

### Comparison of homing-molecule-positive cells in the breast milk of IgAN HC mothers

Next, we analyzed the positivity of selected homing receptors on CD19^+^ cells in breast milk. Because of the low number of EBER^+^ CD19^+^ cells in breast milk in both analyzed groups, we compared the expression of homing receptors on CD19^+^ cells in the breast milk generally.

**Figure 3:**
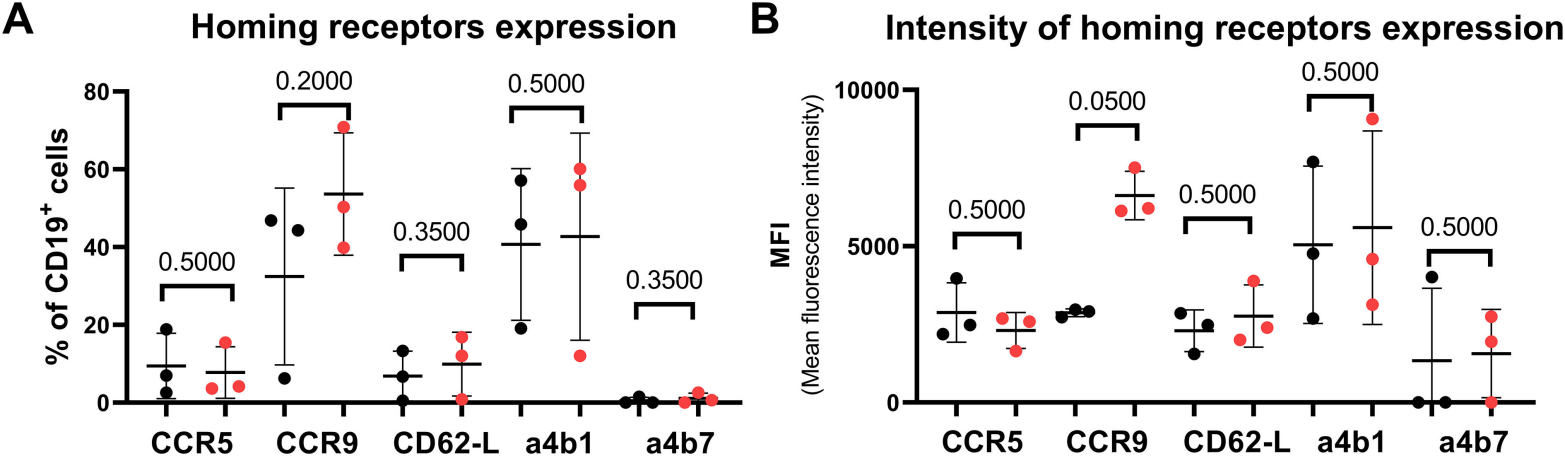
Expression of homing molecules on the surface of CD19^+^ cells. **(A)** Surface expression of CCR5 and CCR9 chemokine receptors, L-selectin, α4β1, and α4β7 integrins on CD19^+^ cells in the breast milk of healthy controls (black) and IgAN mothers (red). **(B)** Surface expression intensity analyses of homing receptors on CD19^+^ B cells in breast milk. Significant differences were detected by using non-paired non-parametric Mann–Whitney test.

Although no significant differences in the percentages of analyzed migration molecules were detected between the breast milk cells of IgAN mothers and healthy controls, their expression corresponds to already published data. The low proportion of CCR5^+^ and L-selectin^+^ cells (both below 10%) may indicate rather homeostatic than inflammatory conditions both in HC and IgAN milk samples (20). There is a low percentage of α4β7^+^ CD19^+^ cells in both analyzed groups. Nevertheless, the expression of α4β7 integrin in breast milk cells is highly controversial because it remains unclear whether α4β7 integrin expression indicates that the cells originate from mucosal immune sites such as GALT or whether it primarily reflects their capacity to migrate to mucosal tissues through interaction with MAdCAM-1 (21). Mainly α4β1 integrins and VCAM-1 primarily mediate homing to mammary tissue for passive immunity transfer via milk (22), and the expression of α4β1 homing receptor on CD19^+^ cells was relatively high in both groups (46% HC vs. 56% IgAN) (**Figure 3A**). The percentage of α4β1^+^ cells in the breast milk of IgAN mothers varies substantially (**Figure 3A**), which could indicate a highly variable migration history of milk α4β1^+^ CD19^+^ cells. The proportion of CCR9^+^ subsets of breast milk CD19^+^ cells was slightly higher in the breast milk samples of IgAN mothers (50%) than that in healthy controls (44%) (**Figure 3A**), although not statistically significant due to the large variability between samples. Because CCR9 is involved in homing to gut mucosa, these cells may have repopulated breast tissue from gut mucosal tissue (19, 23, 24).

Next, we compared the intensity of particular migration molecule expression in CD19^+^ B cells in the breast milk of IgAN patients and healthy controls (**Figure 3B**). While CCR5, CD62-L, α4β1, and α4β7 surface expression was similar in both analyzed groups, the expression of CCR9 in the IgAN group was significantly higher than in the healthy control group (**Figure 3B**). The high CCR9 MFI on breast milk B cells suggests that these cells are strongly mucosa-imprinted lymphocytes with enhanced potential to home to the intestinal mucosa, reflecting the activity of the gut-mammary-immune axis (25–27).

### Gd-IgA1 expression on IgA^+^ cells from breast milk

**Figure 4:**
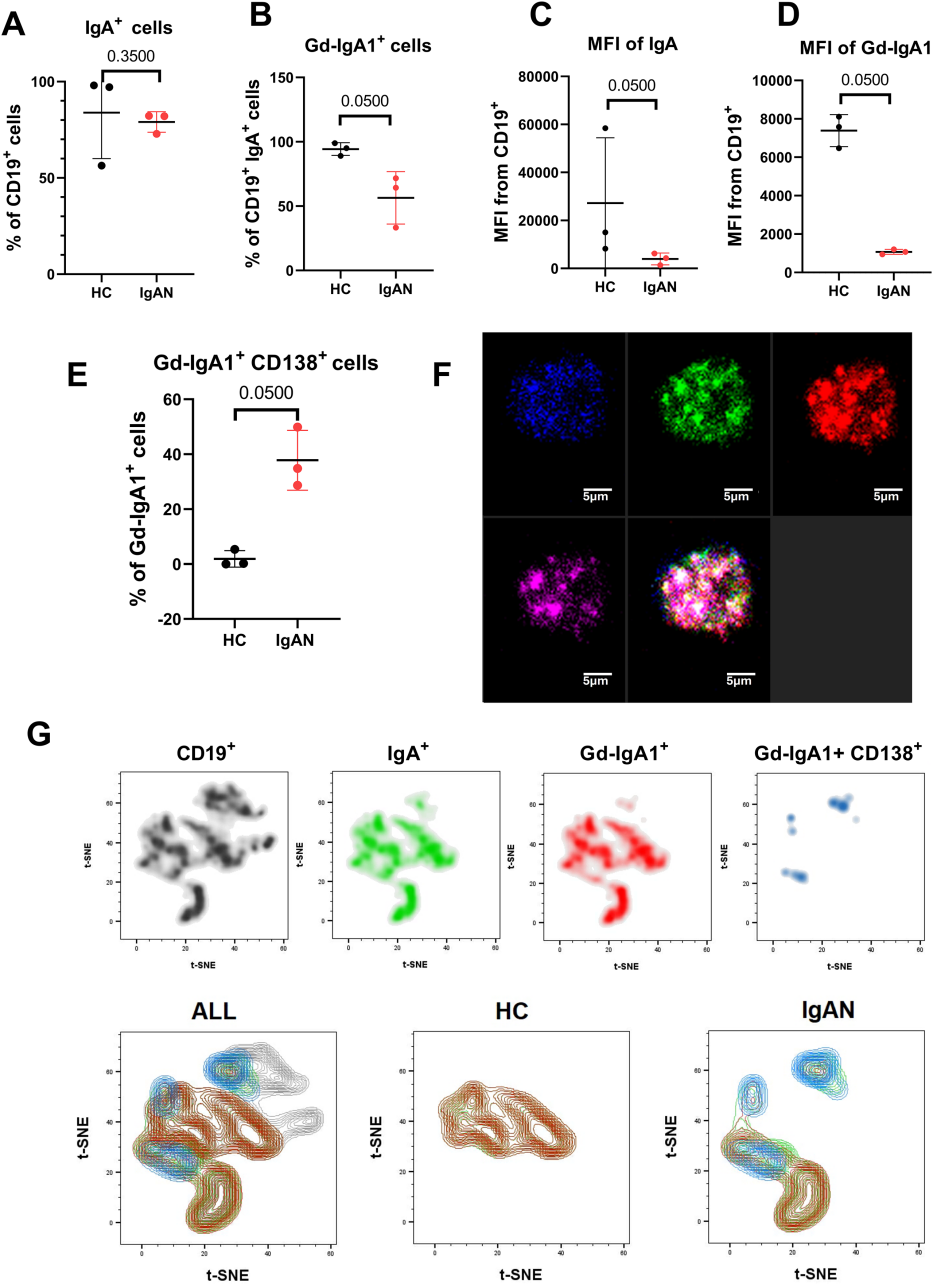
Surface expression of Gd-IgA1 in CD19^+^ sIgA^+^ cell in breast milk. **(A)** Percentage of CD19^+^ IgA^+^ cells in the breast milk of three HC and three IgAN mothers. **(B)** Percentage of surface Gd-IgA^+^ cells in sIgA^+^ CD19^+^ cells isolated from the breast milk of HC (*n* = 3) and IgAN mothers (*n* = 3). **(C)** CD138^+^ population among Gd-IgA1^+^ cells. **(D)** Fluorescence microscopy characterization of the Gd-IgA1^+^ (green) fluorescence pattern on IgA^+^ (blue), CD19^+^ (pink), and CD138^+^ (pink) positive cell. **(E)** Surface IgA expression level on CD19^+^ IgA^+^ cells. **(F)** Surface Gd-IgA expression level on CD19^+^ Gd-IgA1^+^ cells. **(G)** t-SNE analysis of IgA^+^, Gd-IgA1^+^, and CD138^+^ cells in the breast milk of IgAN mothers and healthy controls. The significant values between HC and IgAN in each characterized molecule were calculated by using non-paired non-parametric Mann–Whitney test.

It was reported before that in peripheral blood mononuclear cells (PBMC) the population of surface (s) sGd-IgA1^+^ cells represents about 1% of total sIgA^+^ cells, and this percentage did not differ between the IgAN group and the healthy controls (10). In contrast, in our breast milk samples, the sGd-IgA1^+^ cells are substantially enriched. Interestingly, the percentage of Gd-IgA1^+^ cells in the HC breast milk sIgA^+^ CD19^+^ population is significantly higher than in the IgAN group (94% versus 56%) (**Figure 4B**), whereas the population of sIgA^+^ cells does not significantly differ between both groups (**Figure 4A**). Almost 38% of sGd-IgA1^+^ cells in the IgAN cohort are CD138^+^ (CPC), in contrast to only 1.9% CPC of sGd-IgA1^+^ in HC (**Figure 4E**). The presence of CD19, CD138, IgA, and Gd-IgA co-expression was also shown by fluorescence microscopy (**Figure 4C**). The intensity of surface IgA expression is shown in **Figure 4D**, where a significant increase was detected in the HC group compared to the IgAN group, which corresponds to surface Gd-IgA1 expression (**Figure 4D**). The intensity of surface Gd-IgA1 expression is significantly higher on cells from the breast milk of healthy controls compared to cells from IgAN mothers (**Figure 4F**), which is also confirmed by the visualization of the measured data in **Figure 4G**. The lower expression of surface IgA and Gd-IgA could indicate a higher differentiation state of B cells (28). Differences in quantity and also quality are visualized in multi-dimensional data analysis using t-SNE graphs (t-distributed stochastic neighbor embedding method) (**Figure 4G**). The t-SNE analysis included the following parameters: FSC-A, SSC-A, CD19, IgA, Gd-IgA, and CD138. Several subpopulations are visible in the resulting plots, which is likely because of the relatively low number of B cells in breast milk in general. Nevertheless, this supplementary analysis visually supports the results presented in the graphs. All CD19^+^ cells were characterized for the presence of sIgA^+^ (green), sGd-IgA1^+^ (red), and sGd-IgA1^+^ CD138^+^ CPC (blue). When separated into the HC and IgAN groups, it is clear that HC have more sGd-IgA1^+^ than IgAN, but the phenotype is mostly not corresponding to CPC in contrast to IgAN (**Figure 4G**).

### IgA and Gd-IgA production in the breast milk of IgAN mothers and HC

**Figure 5:**
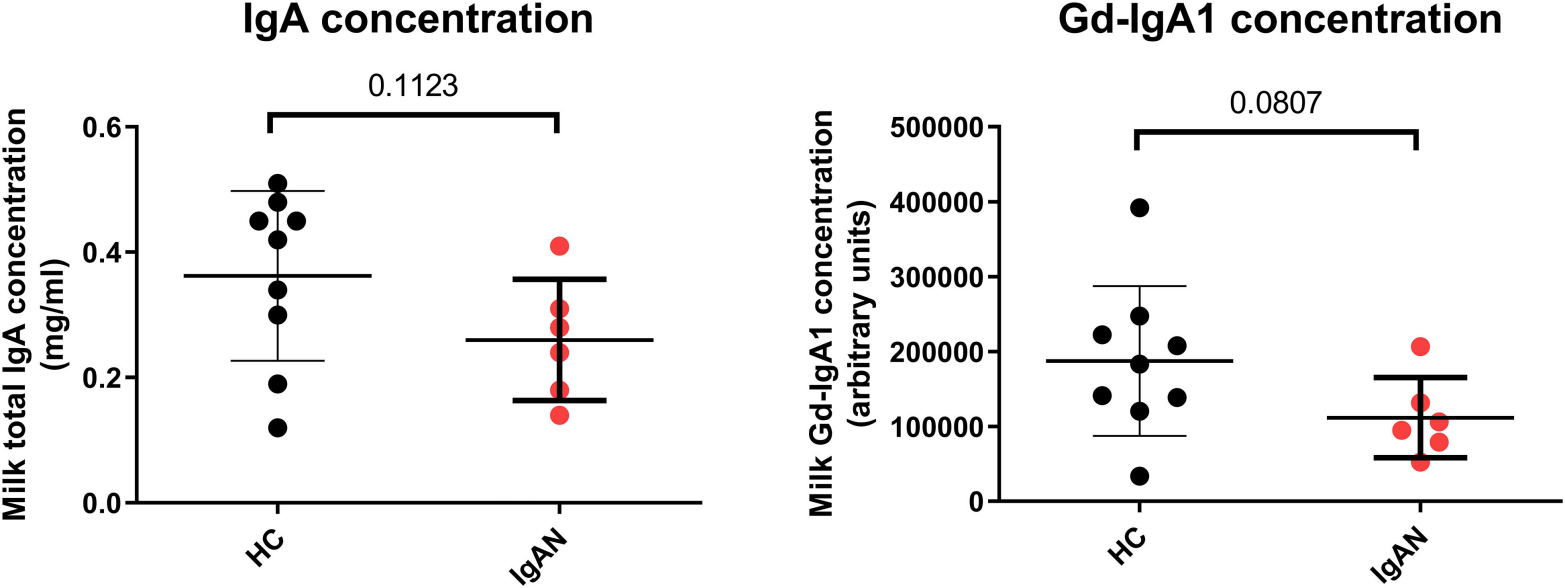
IgA and Gd-IgA1 concentration in the breast milk of IgAN and HC subjects. Comparison of milk levels of IgA and Gd-IgA1 in healthy controls (HC, *n* = 9) and IgAN (IgAN, *n* = 6) breast-feeding mothers. The healthy control’s milk was sampled three times, and those of IgAN mothers were sampled twice. In graphs, means and standard deviations are shown. No significant differences were detected using non-paired *t*-test with Welch’s correction.

Although the populations of sGd-IgA1^+^ cells are significantly higher in HC than in IgAN mother’s breast milk, there are no significant differences in the breast milk IgA and Gd-IgA1 concentration in HC and IgAN mothers (**Figure 5**). These results indicate that the secretion of IgA and Gd-IgA1 into breast milk and the presence of Gd-IgA1^+^ and IgA^+^ cells within the mother’s milk are independent, and secreted immunoglobulins are produced by cells located in the mammalian gland tissues. Cells characterized here are migrating living cells that have potential to be delivered to a newborn.

## Discussion

The composition of breast milk plays a fundamental role in establishing and shaping the mucosal immunity in the newborn (24). While the humoral components of breast milk have been extensively characterized, the cellular compartment remains incompletely understood. Importantly, immune cells derived from breast milk can traverse the infant’s gastrointestinal tract, colonize mucosal tissues, and contribute to the development of primary immune responses. B cells in breast milk may therefore influence susceptibility to immune-mediated diseases, including IgA nephropathy (IgAN), through mechanisms involving mucosal homing, IgA production, and interactions with the developing gut microbiota, although direct causal relationships remain to be defined (13, 24, 29).

Breast milk has been repeatedly reported as a source of EBV-derived nucleic acids, including EBV-encoded RNA (EBER), indicating that the presence of EBER^+^ immune cells at this mucosal site is highly probable (30–32). However, a comparative quantitative and phenotypic analysis of CD19^+^ B cells in breast milk from IgAN mothers and healthy controls has not previously been performed. Given our earlier observation of increased frequencies of EBER^+^ B cells in the peripheral blood of patients with IgAN (8–10), we extended our analysis to B cell populations in breast milk from IgAN mothers and matched healthy controls.

Here we show that consistent with findings in peripheral blood, breast milk from mothers with IgAN contains a significantly higher proportion of EBER^+^ CD19^+^ cells compared with healthy controls. Within the CD19^+^ EBER^+^ compartment, breast milk from IgAN mothers showed a significantly greater enrichment of highly differentiated migrating pre-plasma cells, mirroring alterations observed in peripheral blood B cells and indicating a possible coordinated systemic and mucosal impact driven by EBV infection. The advanced differentiation of B cells toward CPC in IgAN may be driven by an altered cytokine milieu that sustains chronic immune activation. Interleukin-6 (IL-6), a key cytokine promoting plasma cell differentiation, is consistently elevated in patients with IgAN and is detectable at increased levels not only in serum but also in urine, reflecting ongoing inflammatory activity (33–37).

In the context of IgA nephropathy, a direct analysis of IgA^+^ EBER^+^ CD19^+^ cells would be the most disease-relevant approach. However, the frequency of IgA^+^ B cells in breast milk is relatively low, limiting reliable subdivision into EBER-positive and EBER-negative populations, followed by analysis of migratory molecules. Nevertheless, microscopic analysis revealed the presence of cells bearing surface IgA and Gd-IgA1, indicating that IgA-expressing B cells are detectable within the breast milk compartment.

Breast milk B cells exhibit phenotypic features consistent with mucosal origin and share similarities with gut-associated lymphoid tissue (GALT)-related B cells, including the variable expression of α4β7 integrin, CCR9, and low levels of L-selectin (19, 23, 38). However, cell populations with the predominant pattern of α4β1^high^, CCR5^low^, and α4β7^+/−^ expression suggest that these cells are more likely derived from GALT or other mucosal sites rather than actively homing to GALT (19, 23). It has therefore been proposed that CD19^+^ B cells present in breast milk originate from distinct mucosal compartments where they have previously encountered antigen (19, 39).

Lymphocytes in breast milk survive neonatal gastrointestinal transit and migrate to Peyer’s patches, contributing to mucosal immune education (38). These cells show an increased expression of the gut-homing receptor CCR9 (26). Consistently, we observed significantly higher cell surface expression of CCR9 on B cells in breast milk from mothers with IgAN, indicating the retained mucosal homing potential of these B cells. In contrast, the populations of CCR5^+^ or CD62L^+^ were very low, which correspond to published data (19, 20, 23). Higher proportions of populations having α4β1^high^ surface expression with variable α4β7 expression on breast milk leukocytes have been described (19), and the same results have also been observed in our study. This integrin profile suggests a closer relation to GALT-derived B cells than to lymphoid tissues of the salivary glands or bronchial mucosa, where VCAM-1-dependent trafficking pathways predominate. Notably, the functional specificity of α4β7 is context-dependent and influenced by local chemokines and divalent cations, which should be considered when interpreting homing behavior (40–43).

We also observed that breast milk from healthy mothers contains a significantly higher proportion of Gd-IgA1^+^ B cells compared with breast milk from mothers with IgA nephropathy (IgAN). Phenotypic characterization revealed that these cells predominantly exhibit a memory B cell or plasmablast phenotype that was determined by the absence of the CD138 marker and the presence of isotype switched IgA or Gd-IgA1 immunoglobulin on the surface of B cells. In contrast, breast milk from mothers with IgAN was enriched for Gd-IgA1^+^ CD138^+^ CPC, indicating a shift toward terminally differentiated, antibody-secreting cell populations.

The presence of CD19^+^ CD138^+^ cells in breast milk likely represents a transit compartment rather than a terminal effector niche for antibody-secreting cells. These cells are probably on route to effector sites, including the infant gut, where they may contribute to local IgA immunity. The expression of CD19, BCR, and CD138 observed on breast milk B lineage cells supports the concept that breast milk contains a dynamic mixture of memory B cells, plasmablasts, and migrating pre-plasma cells in transit rather than a static population of resident antibody-secreting cells (38, 44, 45). This interpretation is particularly relevant when assessing disease-associated alterations in B cell differentiation and trafficking in IgA nephropathy.

We hypothesize that the increased frequency of galactose-deficient IgA1 (Gd-IgA1)-positive IgA^+^ cells in the breast milk of healthy mothers reflects a physiological mucosal IgA response that is appropriately compartmentalized and functionally regulated, whereas in IgA nephropathy (IgAN) this response is dysregulated and diverted away from the mammary mucosa. However, we are fully aware that further investigations are required to confirm or refute this hypothesis.

In healthy individuals, galactose-deficient IgA1 may be transiently generated at mucosal sites as part of physiological IgA diversification during plasmablast differentiation in response to microbial antigens. Such mucosal IgA responses are tightly regulated and contribute to immune tolerance and commensal-specific immunity rather than systemic immune activation (24). Within the breast milk compartment, Gd-IgA1^+^ IgA^+^ B cells likely represent a controlled mucosal B cell population whose role has not yet been fully elucidated and requires further precise analyses for comprehensive understanding (38, 46).

In contrast, IgA nephropathy (IgAN) is characterized by dysregulated mucosal immune responses, aberrant B cell trafficking, and altered cytokine signaling that promote the excessive systemic release of Gd-IgA1-producing cells rather than their retention within protective mucosal compartments (47–49). Epstein–Barr virus (EBV), which preferentially infects IgA^+^ B cells, may further amplify this process by driving terminal plasma cell differentiation and sustained Gd-IgA1 production. Consequently, Gd-IgA1^+^ cells are underrepresented in the breast milk of mothers with IgAN despite the same proportions of Gd-IgA1^+^ cell populations in IgAN and HC in peripheral blood. Considering all of these data, it remains unclear whether this aberrant localization could promote pathogenic immune complex formation and glomerular deposition in the kidney and also if it could contribute to the exhaustion of protective mucosal IgA responses. Future research will be required to address these and numerous other questions, providing a comprehensive understanding of IgA nephropathy pathology (47, 50).

## Data Availability

The original contributions presented in the study are included in the article/**Supplementary Material**. Further inquiries can be directed to the corresponding author.
