# Supplementary material for: IgA nephropathy-associated breast milk B cell alterations
Source: Front Immunol. 2026 May 8;17:1784598. doi: 10.3389/fimmu.2026.1784598 (PMC13194096; doi:10.3389/fimmu.2026.1784598)
Supplement: Supplementary file 1 [file Table1.docx]

**Supplementary Figure 1.**

**
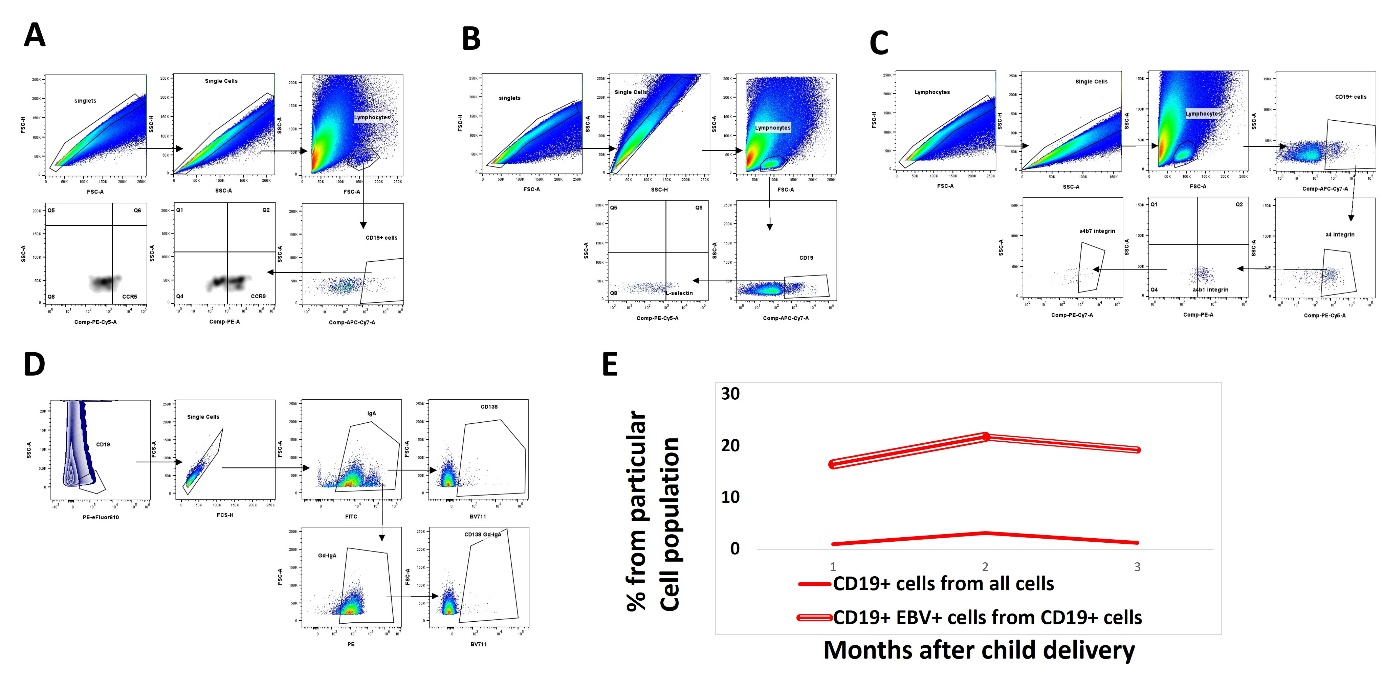
**

**A – C)** Gating strategy of CD19^+^ cells detection. After selecting single cells (FSC-A vs. FSC-H and SSC-A vs. SSC-H), lymphocytes were selected, followed by CD19^+^ cells gating. **A**) CCR5^+^ and CCR9^+^ cells were detected. **B)** L-selectin^+^ cells were gated. **C)** α4 integrin positive cells were gated, followed by β1 and β7 integrin^+^ cells selection. **D)** Gating strategy for detection of IgA^+^ and Gd-IgA1^+^ cells and CD138^+^ CPC. Breast milk cells were gated based on CD19^+^ cell surface expression. Next doublets were eliminated (FSC-A vs FSC-H) and surface IgA^+^ cells were selected. Next Gd-IgA1^+^ cells were detected together with CD138^+^ cell surface marker on IgA^+^ or Gd-IgA1^+^ cells. **E**) Proportions between CD19^+^ and CD19^+^ EBER-infected cells in different sampling time intervals (months after child delivery) remain constant.

**Supplementary Table 1. Detection of EBV antigens from human serum.**

|  | **EBNA1 IgG** | | **VCA IgG** | | **Final results** |
| --- | --- | --- | --- | --- | --- |
|  | **Index** | **Positivity** | **Index** | **Positivity** |  |
| **IgAN** | 265,2519894 | ++ | 191,3917 | + | **seropositive** |
| **IgAN** | 482,1198868 | +++ | 571,9488 | +++ | **seropositive** |
| **HC** | 631,4914832 | +++ | 422,6835 | ++ | **seropositive** |
| **HC** | 576,1703803 | +++ | 497,0346 | ++ | **seropositive** |

IgAN (n = 2) mothers and healthy controls (n = 2) characterized for EBV infected cells in breast milk donored also peripheral blood from where EBV related antigens were determined. All of them were detected as EBV seropositive.

**Supplementary Table 2. Cell counts within individual analyses**

| **Cell counts** | **CD19^+^ cells** | **CD19^+^ EBER^+^** | **CD19^+^ CD138^+^** | **CD19^+^ EBER^+^CD138^+^** |  |  |  |  |
| --- | --- | --- | --- | --- | --- | --- | --- | --- |
| **IgAN** | 445 | 154 | 160 | 110 |  |  |  |  |
|  | 24 | 5 | 11 | 3 |  |  |  |  |
|  | 26 | 8 | 16 | 6 |  |  |  |  |
| **HC** | 28 | 1 | 11 | 0 |  |  |  |  |
|  | 71 | 8 | 4 | 4 |  |  |  |  |
|  | 157 | 18 | 57 | 8 |  |  |  |  |
|  |  |  |  |  |  |  |  |  |
| **Cell counts** | **CD19^+^ cells** | **CD19^+^   L-selectin^+^** | **CD19^+^ cells** | **CD19^+^ CCR9^+^** | **CD19^+^ CCR5^+^** | **CD19^+^ cells** | **CD19^+^ α4β1^+^** | **CD19^+^ α4β7^+^** |
| **IgAN** | 696 | 117 | 910 | 644 | 33 | 25 | 3 | 0 |
|  | 120 | 1 | 8342 | 4198 | 348 | 580 | 324 | 15 |
|  | 150 | 18 | 231 | 92 | 27 | 158 | 95 | 1 |
| **HC** | 60 | 1 | 2083 | 975 | 6 | 28 | 16 | 0 |
|  | 208 | 6 | 1652 | 732 | 53 | 94 | 18 | 0 |
|  | 90 | 8 | 32 | 2 | 115 | 131 | 60 | 2 |
|  |  |  |  |  |  |  |  |  |
| **Cell counts** | **CD19^+^ cells** | **CD19^+^ IgA^+^** | **CD19^+^ IgA^+^ CD138^+^** | **CD19^+^ IgA^+^ Gd-IgA^+^** | **CD19^+^ IgA^+^  Gd-IgA1 CD138^+^** |  |  |  |
| **IgAN** | 81 | 64 | 9 | 45 | 5 |  |  |  |
|  | 7 | 5 | 1 | 4 | 1 |  |  |  |
|  | 86 | 58 | 12 | 35 | 3 |  |  |  |
| **HC** | 132 | 125 | 0 | 123 | 0 |  |  |  |
|  | 14759 | 13389 | 203 | 12677 | 96 |  |  |  |
|  | 2531 | 2188 | 0 | 2172 | 0 |  |  |  |
